# Factors associated with extended remission in neovascular age-related macular degeneration on pro re nata treatment protocol

**DOI:** 10.1136/bjophthalmol-2018-313447

**Published:** 2019-07-13

**Authors:** Tiezhu Lin, Kunny C Dans, Ilkay Kilic Muftuoglu, Amit Meshi, Manuel J Amador-Patarroyo, Lingyun Cheng, William R Freeman

**Affiliations:** 1 Ophthalmology, Jacobs Retina Center, Shiley Eye Institute, University of California San Diego, San Diego, California, USA; 2 Ophthalmology, He Eye Hospital, He University, Shenyang, China; 3 Ophthalmology, Istanbul Training and Research Hospital, Istanbul, Turkey; 4 Ophthalmology, Rabin Medical Center, Petach Tikva, Israel; 5 Ophthalmology, Escuela Superior de Oftalmologia, Instituto Barraquer de America, Bogota, Colombia

**Keywords:** retina, neovascularisation, macula, imaging, treatment medical

## Abstract

**Aim:**

To show the characteristics and outcomes of patients with neovascular age-related macular degeneration (nAMD) who had extended remission (ER) while on a pro re nata (PRN) treatment protocol.

**Methods:**

This was a retrospective case–control study of a consecutive series of patients with nAMD treated with a PRN antivascular endothelial growth factor (anti-VEGF) drug regimen. ER was defined as the absence of haemorrhage, intraretinal/subretinal fluid on optical coherence tomography and leakage on fluorescein angiography for 52 weeks after cessation of anti-VEGF therapy. Matching patients with nAMD who did not achieve ER were included as control group. Cox regression analysis was fitted to identify predictors of time to achieve ER and time to recurrence. A logistic regression analysis of baseline characteristics was used to identify predictors of achieving ER.

**Results:**

Of 830 eyes treated with anti-VEGF monotherapy, 77 (9.2%) eyes achieved ER during a median follow-up of 236 weeks (range 70–525 weeks). Cox regression analysis showed that ER was achieved earlier in eyes with isolated intraretinal fluid (HR, 2.05; 95% CI 1.929 to 4.520; p=0.045) at presentation. Logistic regression analysis showed that type 3 choroidal neovascularisation (OR, 0.090; 95% CI 0.021 to 0.382; p=0.001), thinner choroid (OR, 0.993; 95% CI 0.988 to 0.998; p=0.004) and absence of macular atrophy (OR, 0.233; 95% CI 0.065 to 0.839; p=0.026) at baseline increased the likelihood of achieving ER.

**Conclusion:**

ER is achievable in 9.2% of patients under PRN therapy for nAMD. At presentation with nAMD, anatomical features on retinal imaging may predict the likelihood of achieving ER and a shorter time to achieve ER.

## Introduction

Age-related macular degeneration (AMD) is the major cause of blindness in elderly people in industrialised countries.^1^ The estimated prevalence of late AMD among the elderly people in the USA is 0.8%.^2^ The number of legally blind people in the world is expected to be 2.0 million, and 8.8 million people will have moderate and severe vision loss due to late AMD in 2020.^3^

The advent of antivascular endothelial growth factor (anti-VEGF) agents has improved the clinical outcomes of patients with neovascular AMD (nAMD), as shown in the pivotal Anti-VEGF Antibody for the Treatment of Predominantly Classic Choroidal Neovascularization in AMD (ANCHOR) and Minimally Classic/Occult Trial of the Anti-VEGF Antibody Ranibizumab in the Treatment of Neovascular AMD (MARINA) clinical trials.^4–8^ Numerous other studies such as Comparison of AMD Treatments Trials (CATT), VEGF Trap-Eye: Investigation of Efficacy and Safety in Wet AMD (VIEW) and Lucentis Compared to Avastin Study (LUCAS) have suggested that regular therapy to eliminate subretinal vascular leakage improves visual outcomes. Treatment regimens currently used include pro re nata (PRN) and ‘treat and extend’ (TAE) regimens, which are commonly used to reduce treatment burden for both patients and doctors.^9–13^

The PrONTO study showed similar outcomes between monthly dosing and a PRN regimen, with fewer intravitreal injections over 2 years.^14^ The IVAN study also provided equivalent effect between monthly and as-needed groups at year 1 and year 2.^15–17^ Although the CATT study demonstrated that the mean gain in visual acuity was greater for monthly than for as-needed treatment in year 2, there was no statistically significant difference in visual acuity or morphological outcomes between drug or regimen groups in year 5.^11 18^

A prospective trial of TAE versus monthly dosing of intravitreal ranibizumab for nAMD provided comparable results between the two groups at 1 year.^19^ Gupta *et al* 20 also reported that TAE regimen showed favourable visual outcomes with fewer patient visits, fewer treatments and lower medical costs compared with monthly injections, but more injections compared with the PrONTO study.

Compared with a PRN treatment regimen, the maximal length of treatment interval between injections using the TAE regimen is typically 12 weeks.^20 21^ Therefore, patients have to continue to receive maintenance injections at least every 12 weeks regardless of disease activity when using TAE regimen. In our experience using PRN therapy, some patients show absent retinal exudation for much longer intervals after injections are discontinued and may not need retreatment for intervals well over the typical 3-month TAE maximum interval.^22^ For these patients, the PRN regimen would be much less burdensome than the TAE regimen^23^ and would also decrease cost and the risk of complications. In addition, a true PRN regimen is the only way to determine if some eyes will not require ongoing maintenance treatment. It has been suggested that, in a small group of patients who met predefined exit criteria, treatment may be discontinued with a low incidence of recurrences.^24^ Similarly, in the CATT 5-year follow-up study, 96 patients (14.8%) received no treatments between the end of the 2-year clinical trial and the 5-year CATT follow-up study visit.^11^ However the CATT study terminated after 2 years. The IVAN study likewise found that the PRN strategy was non-inferior to monthly treatment.^17^ In another retrospective study of the PRN treatment in Japan, 81 of 236 eyes (34.3%) and 35 of 139 eyes (25.2%) had no recurrence after three loading anti-VEGF injections in year 1 and year 2, separately.^25^ However, Asian eyes may have different responses from the typical US population.

Previously, we reported the results of a small study evaluating 6-month remissions without anti-VEGF therapy and found certain predictors of this duration of remission.^22^ Our purpose in this study was to evaluate a larger population with longer follow-up, and to evaluate the predictors and prevalence of over 1-year (extended) remission in eyes treated with PRN anti-VEGF therapy to better understand our therapies and provide guidance for treatment regimens.

## Methods

### Study design

This was a retrospective chart review of treatment-naïve patients with newly diagnosed nAMD seen at the Jacobs Retina Center and Shiley Eye Institute, University of California San Diego.

### Study population

The charts of all patients with nAMD seen over a 10-year period between 2008 and 2018 were reviewed. The inclusion criteria of the remission group were (1) new-onset nAMD at presentation, (2) age >50 years, (3) three consecutive monthly bevacizumab or ranibizumab (Genentech, San Francisco, California, USA) injections followed by as-needed treatment regimen, (4) remission time ≥52 weeks at any time point during follow-up, and (5) best-corrected visual acuity (BCVA) of 20/160 or better at the final visit. Eyes were excluded in the presence of (1) a central disciform scar, (2) central geographic atrophy, (3) concomitant ocular diseases (ie, diabetic retinopathy, glaucoma, retinal vein occlusion, uveitis or epiretinal membrane), (4) choroidal neovascularisation (CNV) secondary to causes other than AMD (ie, myopic CNV, angioid streaks or other secondary CNV), (5) a history of vitrectomy, laser treatment or photodynamic therapy (PDT), or (6) monocular status. Only binocular patients were included in this study, as continuous monthly or every-8-week intravitreal anti-VEGF injections were given to monocular patients.

A group of patients with new-onset nAMD treated with as-needed anti-VEGF therapy without extended remission (ER) matched for age, gender, ethnicity, baseline BCVA and follow-up time were included as the control group. Baseline presentation features were compared between the remission and the control groups.

### Ophthalmological examination and retinal imaging

All patients underwent a complete ophthalmological examination and retinal imaging at baseline (on diagnosis of nAMD) and at each follow-up visit, including BCVA measurement using the ETDRS chart, slit-lamp biomicroscopy and indirect ophthalmoscopy through dilated pupils.

Retinal imaging was performed, including fluorescein angiography (FA) and high-resolution spectral domain optical coherence tomography (SD-OCT) using a device coupled with a simultaneous confocal scanning laser ophthalmoscopy (cSLO) (Spectralis HRA+OCT, Heidelberg Engineering, Carlsbad, California, USA). As a routine imaging protocol, raster SD-OCT scans along with horizontal and vertical B-scans through the fovea, as well as through other areas where CNV activity was most pronounced, were acquired.

### Treatment protocol

Intravitreal anti-VEGF treatment was started when the SD-OCT showed exudation involving the fovea, with either intraretinal or subretinal fluid (IRF/SRF) accompanied by leakage on FA. Pigment epithelial detachments (PED) in the absence of IRF or SRF were not treated.

The standard protocol employed for the treatment of nAMD and macular fluid was three loading intravitreal injections of bevacizumab (1.25 mg/0.05 mL) or ranibizumab (0.5 mg/0.05 mL) given at 4-week intervals and under SD-OCT and FA guidance. Injections were continuously given at 4-week intervals until the retina became completely dry, after which an observation phase was initiated. Patients were followed at 4, 8 and 12 weeks from the last injection, followed by a visit every 24 weeks thereafter. Patients were given precautions regarding warning signs, including decline in vision and worsening metamorphopsia, with instructions to follow up immediately if any of these are noted.

The same treatment regimen was resumed when there was evidence of recurrence of exudation as we have previously reported.^25 26^ Recurrence was defined as new IRF and/or SRF on SD-OCT and leakage on FA accompanied by symptoms of worsening, including decline in ETDRS BCVA or presence of metamorphopsia. Resolution was defined as the absence of leakage on FA and complete absence of IRF and SRF on SD-OCT.

Eyes that demonstrated treatment resistance or non-response to bevacizumab or ranibizumab were shifted to aflibercept (Regeneron, Tarrytown, New York, USA) 2.0 mg/0.05 mL since 2012, when aflibercept became available. Treatment resistance was defined as having multiple recurrences (minimum of two recurrences after the eyes have been completely dry following a series of at least 3-monthly injections per treatment cycle), or persistence of exudation (poor response to monthly ranibizumab or bevacizumab injections for at least 5 months) as evident on clinical examination and on imaging studies (leakage on FA, or fibrovascular PED with IRF and/or SRF on SD-OCT) while on monthly ranibizumab or bevacizumab monotherapy.^25^ A dose-escalating regimen of aflibercept 2.0 mg/0.05 mL was given every 8 weeks and escalated to every 4 weeks if needed, depending on clinical response.

Intravitreal injections were carried out under aseptic conditions in the clinic. Preservative-free lidocaine gel was instilled in the superior fornix at least 5 min prior to the injection. A lid speculum was placed and 5% povidone iodine solution was instilled twice on the conjunctiva prior to injection. The lidocaine gel was displaced by the povidone iodine. The pars plana was marked externally 3.5–4.0 mm from the limbus, depending on the lens status. The intravitreal injection was performed using a 30-gauge needle in the superotemporal or superonasal quadrant. Following the injection, eyes were thoroughly washed with sterile balanced salt solution. Topical antibiotic drops were not prescribed after the procedure.

### Data collection

The baseline demographic data collected from each patient’s record included age, gender, ethnicity, laterality of the involved eye, BCVA, lens status and duration of follow-up. Imaging parameters at baseline were measured by two experienced retina specialists (TL and KCD), taking note of the CNV type (type 1: subretinal pigment epithelium [RPE]; type 2: subretinal; type 3: retinal angiomatous proliferation (RAP)), CNV location (subfoveal, juxtafoveal or extrafoveal), CNV lesion size, central foveal thickness, subfoveal choroidal thickness, fluid type (isolated SRF, isolated IRF, or combined SRF and IRF), presence of outer retinal tubulation, presence of macular atrophy, posterior vitreous status (incomplete or complete posterior vitreous detachment) and PED type (serous, drusenoid or fibrovascular). CNV lesion size was measured as the maximum size of hyperfluorescence on late-phase FA. Macular atrophy was defined as loss of outer retinal layers and RPE with signal hypertransmission to the choroid in the macula. All measurements were done using the calliper function of the Spectralis’ built-in software.

### Statistical analysis

The Shapiro-Wilk test was used to test normality of distribution. The remission and control groups were analysed together in a logistic regression model to identify baseline anatomical factors that are predictive of ER. ETDRS visual acuities were converted to logarithm of the minimum angle of resolution (logMAR) for statistical analysis. Kaplan-Meier survival curves were generated for survival analysis. Univariate and multivariate Cox proportional hazard models were fitted for the time to achieve remission and time to recurrence. Significant variables obtained using univariate analysis were selected for the multivariate Cox regression. P values represent the results for two-sided tests, with values less than 0.05 considered statistically significant. Statistical analyses were conducted using SPSS V.24.0.

## Results

A total of 830 eyes of 558 patients with nAMD were identified during the study period. Of these, 77 eyes (9.2%) of 70 patients had ER at some time point during a median of 236 weeks (range 70–525) follow-up. A random sample of 84 eyes of 70 patients were included as a matched control group. The baseline characteristics are summarised in table 1. The groups were matched by age, gender, ethnicity, baseline visual acuity and duration of follow-up.

**Table 1:** Demographic characteristics of the study participants

	Remission group (n=77)	Control group (n=84)	P value
Age, mean±SD	79.99±8.43	80.1±7.99	0.105
Female, n (%)	45 (58.4)	62 (61.9)	0.605
Ethnicity Caucasian, n (%)	67 (87.0)	72 (85.7)	0.805
Baseline BCVA, mean logMAR±SD (≅Snellen equivalent)	0.40±0.27 (20/50)	0.38±0.27 (20/48)	0.771
Duration of follow-up, median weeks (range)	236 (70–525)	222 (66–508)	0.104

BCVA, best-corrected visual acuity; logMAR, logarithm of minimum angle of resolution.

**Figure 1:**
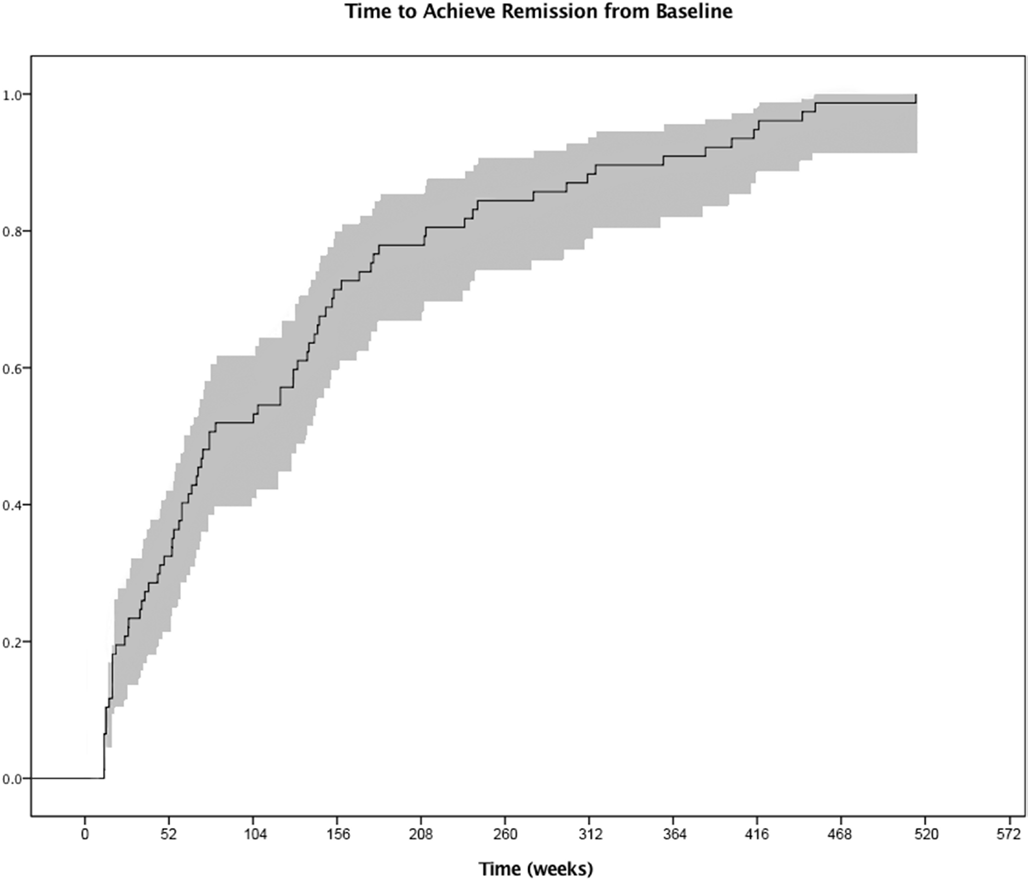
Kaplan-Meier curve showing the time to achieve extended remission from baseline.

**Figure 2:**
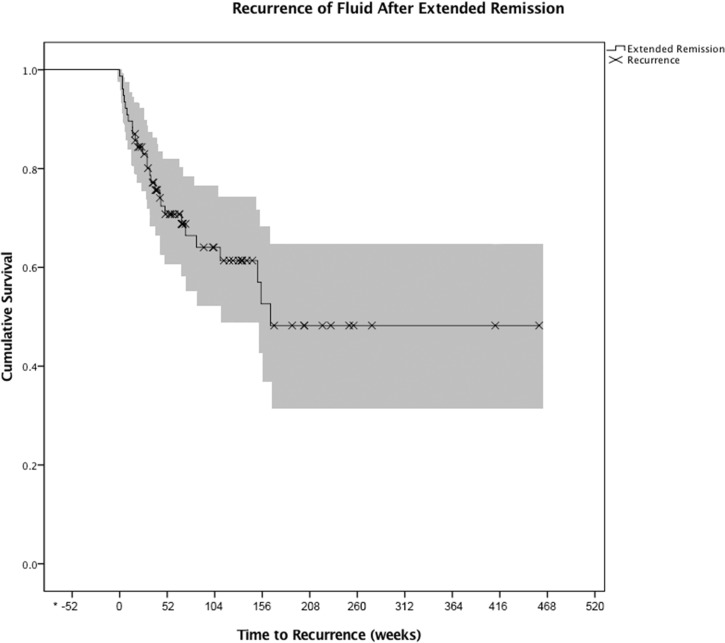
Kaplan-Meier curve of the survival time to recurrence after the last intravitreal injection.

**Figure 3:**
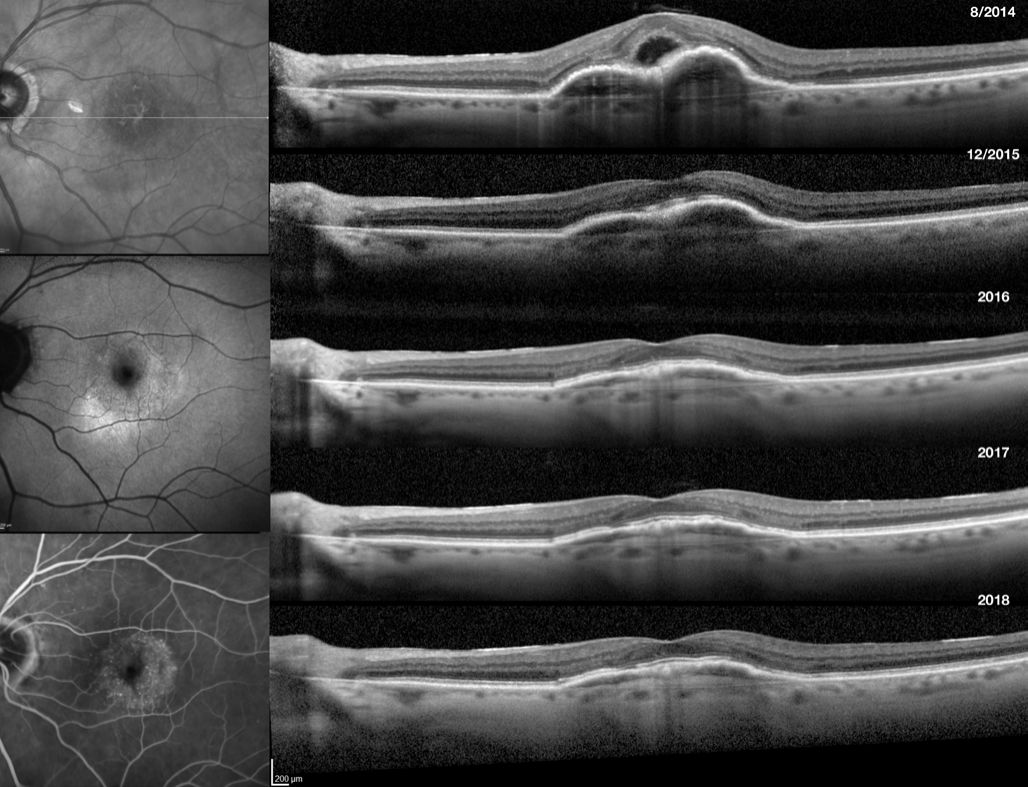
Imaging tests of the left eye in an 82-year-old woman with new-onset nAMD. Visual acuity at presentation was 20/40. Baseline imaging confirmed the diagnosis of nAMD. Infrared photo (top left) showed RPE changes accompanied by a PED in the macula with corresponding mixed increased and decreased autofluorescence signal on FAF (middle left). Leakage was evident on late-phase FA (bottom Left). Horizontal sectional SD-OCT across the fovea at baseline (top right) showed subretinal fluid, subretinal hyper-reflective material and pigment migration overlying a large fibrovascular PED involving the fovea. Monthly intravitreal anti-VEGF treatment was initiated with bevacizumab (#9) and subsequently aflibercept (#5) until the retina was completely dry (sub-top right). The retina remained dry throughout the remainder of follow-up (middle-bottom right), a total duration of remission of 117 weeks. BCVA at the final visit was 20/25. BCVA, best-corrected visual acuity; FA, fluorescein angiography; FAF, fundus autofluorescence; nAMD, neovascular age-related macular degeneration; PED, pigment epithelial detachment; RPE, retinal pigment epithelium; SD-OCT, spectral domain optical coherence tomography; VEGF, vascular endothelial growth factor.

In the remission group, the median time needed to achieve ER after the initial anti-VEGF injection was 77 weeks (range 12–466) (figure 1). The median number of anti-VEGF injections needed to achieve ER was 10 (range 3–56). Forty-one of the 77 eyes (53.3%) were receiving bevacizumab (40) or ranibizumab (1) prior to achieving ER, and 36 (46.8%) were receiving aflibercept injections prior to achieving ER. Thirty-two per cent of eyes (25 eyes) achieved ER during the first year of presentation, 19.5% of eyes (15 eyes) at year 2, 19.5% of eyes (15 eyes) at year 3, and 28.5% of eyes (22 eyes) after more than 3 years of follow-up. The Kaplan-Meier analysis showed a median duration of total remission of 217 weeks (range 52–507 weeks) (figure 2). Sixty-four per cent remained dry and free of exudation at the final visit. Figure 3 illustrates the clinical picture of a patient who achieved ER.

Twenty-eight (36.4%) eyes recurred after a median remission period of 82 weeks (range 52–217). Of these recurrences, five (6.5% of remission group, or 17.9% of recurrences) had a haemorrhagic component, two of which were dense and involved the fovea, and the rest were thin and extrafoveal. There was a decline in vision from a median of 0.25 logMAR (range 0.0–0.9) (SE≅20/36) to 0.32 logMAR (range 0.0–1.0) (SE≅20/42). This change was not statistically significant (p=0.330).

Visual acuity remained stable in the remission group at a median of 0.32 logMAR (range 0–1.0) (Snellen≅20/42) from baseline to 0.30 logMAR (range 0–1.0) (Snellen≅20/40) at the final visit (p=0.831). On the other hand, the control group demonstrated a loss of one line from a baseline of 0.30 logMAR (range 0–1.0) (Snellen≅20/40) to 0.40 logMAR (range 0–3.0) (Snellen≅20/50) at the final visit (p=0.031).

Of eyes that achieved early ER, that is, within the first year of diagnosis and treatment initiation, the median change in visual acuity from baseline to final visit was 0.004 logMAR (range −0.50 to 0.20) (p*=*0.918). The rest of the eyes showed a median of 0.05 logMAR (range −0.40 to 0.50) decline in vision between baseline and end of study (p=0.03).

Univariate and multivariate Cox regression analyses were performed to identify factors for time to achieve ER and the time of remission to recurrence. In the multivariate Cox regression analysis, the presence of isolated IRF at baseline predicted a shorter time to achieve ER (2.05-fold faster, HR, 2.05; 95% CI 1.929 to 4.520; p=0.045) compared with eyes with combined IRF and SRF. Multivariate analysis also showed a trend for older patients needing longer time to achieve ER (HR, 1.035; 95% CI 0.995 to 1.076; p=0.082). There were no significant covariates associated with duration of remission in the Cox regression analysis.

Table 2 summarises the results of the logistic regression analysis. Eyes with type 3 CNV had an 11.111-fold increased likelihood (OR, 0.090; 95% CI 0.021 to 0.382; p=0.001) of achieving ER compared with eyes with type 2 CNV. The presence of macular atrophy at baseline reduced the probability of achieving ER by 4.292-fold. Every micrometre decrease in subfoveal choroidal thickness increased the likelihood of achieving ER by 0.7% (OR, 0.993; 95% CI 0.988 to 0.998; p=0.004).

**Table 2:** Logistic regression analysis of clinical characteristics of remission and control groups at baseline

Variables	OR (95% CI)	P value
CNV type		
Type 3	1.0000	
Type 1	0.000 (0.000 to 0.000)	0.999
Type 2	0.090 (0.021 to 0.382)	0.001
Fluid type		
SRF/IRF combined	1.0000	
Isolated IRF	0.665 (0.217 to 2.033)	0.151
Isolated SRF	5.397×10^9^	0.999
Posterior vitreous status		
Complete PVD	1.0000	
No PVD	9.171 (0.955 to 88.099)	0.055
Incomplete PVD	0.974 (0.303 to 3.123)	0.964
PED		
Fibrovascular	1.0000	
Serous	4.154 (0.312 to 55.308)	0.281
Drusenoid	1.559 (0.242 to 10.034)	0.64
CFT	0.999 (0.996 to 1.003)	0.688
SCT	0.993 (0.988 to 0.998)	0.004
Mean lesion size	0.962 (0.854 to 1.083)	0.519
ORT		
Absent	1.0000	
Present	0.247 (0.053 to 1.157)	0.076
Macular atrophy		
Absent	1.0000	
Present	0.233 (0.065 to 0.839)	0.026

CFT, central foveal thickness; CNV, choroidal neovascularisation; IRF, intraretinal fluid;ORT, outer retinal tubulation; PED, pigment epithelium detachment; PVD, posterior vitreous detachment; SCT, subfoveal choroidal thickness; SRF, subretinal fluid.

## Discussion

In the current study, we report the outcomes of the largest cohort of patients with nAMD that achieved ER undergoing anti-VEGF therapy by PRN regimen yet presented.

We found that 9.2% of eyes with nAMD may achieve ER using a PRN treatment regimen. This is only slightly less than our previous report of 11.6% of PRN-treated eyes with nAMD achieving remission of over 6 months.^22^ The CATT extension study reported that 14.8% of eyes did not receive any treatment between the end of years 2 and 5, although follow-up was incomplete.^11^ In our study, about 10% of eyes remained dry without anti-VEGF injections for a median period of 217 weeks in the PRN regimen and visual acuity was maintained. Moreover, of those achieving an ER, 71% did so within the first 3 years since the onset of the disease. Compared with fixed monthly and TAE regimens, the PRN schedule may have the advantage of permitting cessation of therapy for long periods of time in some patients.^26^ An increased incidence of vision-threatening subretinal haemorrhage was associated with the PRN treatment strategy compared with TAE.^27^ Monocular patients were not included in this study as this group of patients received a fixed dosing schedule of aflibercept given every 8 weeks.

Our study showed that the presence of isolated IRF predicted a shorter time to achieve ER. Interestingly, IRF has been reported to be resolved rapidly with ranibizumab compared with SRF. Possible explanations might be that anti-VEGF medicine rapidly diminishes active neovascular leakage into the retina, and that the intraretinal bioavailability of the drug is rapid and efficient.^28^

We found that eyes with RAP had an 11-fold greater likelihood of achieving ER compared with type 2 CNV. The CATT study group reported that eyes with RAP were less likely to have fluid at 1 and 2 years after initiation of anti-VEGF therapy, but were also more likely to develop retinal atrophy.^29^ Multiple reports describe intraretinal cystoid changes with fluid as a characteristic optical coherence tomography feature of RAP lesions.^30 31^ Moreover, thinner choroid also increased the chance of achieving ER. Prior studies have also suggested these characteristics are associated with the ability to discontinue anti-VEGF therapy for over 6 months.^22^ Interestingly, we found that the presence of macular atrophy at baseline decreased the probability of achieving ER in this study.

The limitations of this study are its retrospective nature and the relatively small study population. We performed the statistical analysis by eye rather than by patient because the rate of bilateral inclusion was only 15% of the total population, which has a low likelihood of introducing bias. We also did not include the last anti-VEGF agent used prior to ER because the decision to switch to aflibercept depended on each patient’s response to bevacizumab or ranibizumab, and the time at which the switch was done varied by clinical response as well. It is important to note that we excluded the risk factors that would make the results biased, including vitrectomy, PDT and laser treatment. We also excluded large subretinal haemorrhage to reduce the risk of including poor prognosis eyes.^32^ In AMD, a vision of 20/200 is usually associated with a disciform scar or central geographic atrophy. These two conditions being present in an eye makes the treatment approach less aggressive. We only included patients with a final visual acuity of 20/160 or better to ensure that the decision to observe was because the disease was truly inactive and not because of less aggressive management due to the poor visual prognosis associated with a disciform scar or central atrophy.

In conclusion, 9.2% of eyes with nAMD would have ER in the PRN regimen. Seventy-one per cent of eyes that achieve ER do so within the first 3 years from baseline. Eyes with isolated IRF at baseline achieve ER faster. The presence of RAP, thinner choroid and absence of macular atrophy at baseline are associated with the likelihood of achieving ER. Nonetheless, because the majority of patients need ongoing treatment, patients should be counselled regarding the need for regular monitoring for early detection of recurrence.
